# A Comprehensive Automated 3D Approach for Building Extraction, Reconstruction, and Regularization from Airborne Laser Scanning Point Clouds

**DOI:** 10.3390/s8117323

**Published:** 2008-11-17

**Authors:** Peter Dorninger, Norbert Pfeifer

**Affiliations:** 1 Christian Doppler Laboratory “Spatial Data from Laser Scanning and Remote Sensing”, Institute of Photogrammetry and Remote Sensing, TU Vienna, Guβhausstraβe 27-29, A-1040 Vienna, Austria; 2 Institute of Photogrammetry and Remote Sensing, TU Vienna, Guβhausstraβe 27-29, A-1040 Vienna, Austria. E-mail: np@ipf.tuwien.ac.at

**Keywords:** building modeling, building outline, segmentation, planar faces, regularization

## Abstract

Three dimensional city models are necessary for supporting numerous management applications. For the determination of city models for visualization purposes, several standardized workflows do exist. They are either based on photogrammetry or on LiDAR or on a combination of both data acquisition techniques. However, the automated determination of reliable and highly accurate city models is still a challenging task, requiring a workflow comprising several processing steps. The most relevant are building detection, building outline generation, building modeling, and finally, building quality analysis. Commercial software tools for building modeling require, generally, a high degree of human interaction and most automated approaches described in literature stress the steps of such a workflow individually. In this article, we propose a comprehensive approach for automated determination of 3D city models from airborne acquired point cloud data. It is based on the assumption that individual buildings can be modeled properly by a composition of a set of planar faces. Hence, it is based on a reliable 3D segmentation algorithm, detecting planar faces in a point cloud. This segmentation is of crucial importance for the outline detection and for the modeling approach. We describe the theoretical background, the segmentation algorithm, the outline detection, and the modeling approach, and we present and discuss several actual projects.

## 1. Introduction

Three dimensional modeling of a city scape requires that individual buildings are represented, next to urban vegetation [1], streets [2], and other objects of the city infrastructure such as watercourses [3], power supply lines [4], and individual objects like street signs or fountains. A Digital Surface Model (DSM) derived from point clouds acquired by Airborne Laser Scanning (ALS) [5] or stereo-photogrammetry [6, 7] already represents buildings. While such models can be generated easily and automated, they represent the approximate roof shapes without generalization and without distinguishing between individual buildings on the one hand and between buildings and other objects like ground and vegetation on the other hand. Visualizations, noise modeling, or interactive measurements are some applications, that can be applied to such city models. By providing building or building block outlines, e.g. from cadastral maps, such models can be enhanced and surface models can be generated for individual buildings or blocks. These models do not allow to distinguish between individual roof faces, nor between roof and dormers or other objects. Furthermore, artifacts of data acquisition, caused e.g. by occluded areas, sampling distance, or remaining geo-referencing errors, are features of such models. Moreover, vertical walls may appear slanted. Nonetheless, geometric parameter like volume and area of complete buildings or area, inclination, and aspect of individual roof faces can be determined automatically and used as input for further analysis.

To increase the reliability of the building models as well as the range of possible applications, additional knowledge on buildings has to be incorporated into the modeling process. Typical assumptions are to define walls as being vertical and roofs as being a composition of planar faces. This leads to an idealization of the buildings. The transition zone of two neighboring roof faces, for example, becomes a straight line defined by the intersection of two roof planes.

The generation of reliable and accurate building models from laser scanning data requires a number of processes. These are building detection, outline extraction, roof shape reconstruction, model generation and regularization, and finally, model quality analysis. The majority of available literature concentrates on individual aspects only. For example, [8–15] describe building region detection in rasterized laser scanning data and [16, 17] describe roof reconstruction in laser scanning point clouds with known building boundaries. Approaches considering detection and reconstruction are presented e.g. by [18] and [19]. The reconstructed models presented in these two references are, however, restricted. In both cases DSM data of relatively low density is processed. This does not allow for exact positioning of building outlines and prevents the reconstruction of small roof features. Furthermore, in the latter reference the complexity of building models is restricted to a composition of predefined building parts.

Our contribution is to present an approach for automated generation of building models from ALS, comprising the entire sequence from extraction to reconstruction and regularization. It is applicable to point clouds of a density of about two points per m^2^, which is state of the art for capturing built-up areas, but it is suited also for high density point clouds with some ten points per m^2^. It uses the point cloud directly, which avoids a loss in precision because of rastering and mixing of vegetation and roof overhangs [20]. In Section 2. the state of the art in building detection and reconstruction is summarized. In Section 3. the theoretical aspects of our approach are presented. In Section 4. the whole workflow from the point cloud to the final building model is described and results are discussed in Section 5..

## 2. Related work

*Building detection* is often performed on resampled (i.e. interpolated) grid data, thus simplifying the 3D content of ALS data to 2.5D. Roughness measures, i.e. local height variations, are often used to identify vegetation. Open areas and buildings can be differentiated by first computing a Digital Terrain Model (DTM) with so-called filtering methods [21, 22]. Thereafter, a normalized Digital Surface Model (nDSM) is computed by subtraction of the DTM from the DSM, hence representing local object heights [8–12]. High objects with low roughness correspond to building areas. Other approaches identify blobs in the DSM, based on height jumps, high curvature, etc. [13–15, 23].

*Building boundaries* are the intersection of the buildings with its surroundings, in general the terrain. If not available (e.g. cadastre), they need to be derived from the given point cloud data. Typically, the building boundary generation is initiated by detecting a coarse approximation of the outline, followed by a generalization and a regularization [24, 25].

A *segmentation* allows for a decomposition of a building as represented in a laser scanning point cloud into planar faces and other objects. This requires the definition of a homogeneity criterion according to which similar items (e.g. points) are grouped. As homogeneity criterion, approximate height similarity or/and approximate normal vector similarity are commonly used. Determination of planar faces for roof modeling from point clouds acquired from airborne platforms is studied in [26–28]. To reduce the complexity of the problem and to increase the performance of the implementation, again, 2.5D grid representations are commonly used instead of the original points. This requires the definition of a reference direction (e.g. the vertical *z*-axis) to resample the given points to a regular grid defined as a scalar function over the horizontal *xy*-plane. Thus, only one distinct height value can be assigned to an arbitrary pair of *xy*-coordinates. Advantages of 2.5D approaches are a possible reduction of the amount of input data and the implicitly defined neighborhood by means of the grid representation. By contrast, for processing original point clouds, such a neighborhood (e.g. for the estimation of normal vectors) has to be defined explicitly (e.g. [20]). Unfortunately, the grid resampling process introduces smoothing effects especially at sharp surface structures. Segmentation approaches based on range images suffer from these restrictions as well (e.g. [8], [9]). By contrast, many approaches described for processing 3D point clouds acquired from terrestrial platforms are designed to operate in 3D space (e.g. [10], [11]). An approach for ALS point clouds segmentation in 3D is suggested by [29].

For *building reconstruction* two fundamentally different approaches can be distinguished: model driven and data driven methodologies. In model driven methods a predefined catalog of roof forms is prescribed (e.g. flat roof, saddle back roof, …). The models are tested and the one with the best fit is chosen [27, 30]. This is especially appropriate for low point densities. An advantage is that the final roof shape is always topologically correct. A disadvantage is, however, that complex roof shapes cannot be reconstructed, because they are not included in the catalog. In data driven methods the roof is “reassembled” from roof parts found by segmentation algorithms. The result of the segmentation process are sets of points, each one ideally describing exactly one roof face. Some roof elements (e.g. small dormers, chimneys, ⋯) may not be represented. The challenge is to identify neighboring segments and the start and end point of their intersection. [16] partly avoids this problem by partitioning the given ground plan and finding the most appropriate (in some cases: nearest) plane segment to each partition.

## 3. Theory

The basic assumption is that a point cloud representing a single building can be decomposed into segments which describe planar patches. These patches are used for subsequent 3D model generation. Hence, this segmentation is of crucial importance for the reliability of the modeling approach and will be, therefore, discussed in detail in this section. Furthermore, we give a detailed description of building extraction also relying on the same point cloud segmentation. Finally, the determination of regularized building outlines from point clouds and the building model generation process are described.

### 3.1. Segmentation initialized by hierarchical clustering

We assume that points belonging to the same planar region have similar local regression planes. Therefore, to determine such planar regions, each point is represented by its local regression plane, which in turn is described by its normal vector and intercept (cf. Eq. 1). In order to distinguish between points belonging to different planar faces, we compute a metric between their corresponding regression planes in a 4D feature space, as explained next. A plane in 3D Cartesian coordinates is defined by

(1) $$0=a_{0}+a_{1}x+a_{2}y+a_{3}z$$

with (*a*_1_, *a*_2_, *a*_3_)^T^ representing the local unit normal vector of the plane and *a*_0_, the normal distance of the plane to the origin of the coordinate system. [31] describe a solution for clustering based on the Gaussian sphere, as the unit normal vectors of points belonging to a plane will occur as a point cluster on this sphere. To separate parallel planar faces, the point cloud has to be analyzed separately in object space. [32] describe another solution. It is a 2.5D-approach and therefore restricted to planes which are not parallel to the *z*-axis. Such planes can be written in the form

(2) $$z=u_{0}+u_{1}x+u_{2}y\;\text{with}\;u_{i}=a_{i}/a_{3}$$

i.e., using homogeneous plane coordinates *U* = (*u*_0_, *u*_1_, *u*_2_, −1. Thus (*u*_0_, *u*_1_, *u*_2_) are affine coordinates in the resulting affine space. A Euclidean metric is introduced in this affine space and the deviation between two planes is measured within a rectangular area of interest Γ, defined by the extension of the object. To determine the distance *d* between two planes *U* = (*u*_0_, *u*_1_, *u*_2_, −1 and *V* = (*v*_0_, *v*_1_, *v*_2_, −1, they choose the Lebesgue measure of the squared differences:

(3) $$d_{\Gamma }{\left(U,V\right)}^{2}=\int_{\Gamma }{\left((u_{0}-v_{0})+(u_{1}-v_{1})x+(u_{2}-v_{2})y\right)}^{2}\;\text{dxdydz}$$

In order to overcome the 2.5D restrictions of this method, we replaced the homogeneous plane definition of Eq. 2 with the 3D definition given in Eq. 1. Additionally, the planar integral (*dxdy* times) defined in Eq. 3 is replaced by a volumetric integral (*dxdydz* times). Thus, for the determination of a 3D distance measure between two planes *A* = (*a*_0_, *a*_1_, *a*_2_, *a*_3_) and *B* = (*b*_0_, *b*_1_, *b*_2_, *b*_3_), Eq. 3 is extended to

(4) $$d_{\Gamma }{\left(A,B\right)}^{2}=\int_{\Gamma }{\left((a_{0}-b_{0})+(a_{1}-b_{1})x+(a_{2}-b_{2})y+(a_{3}-b_{3})z\right)}^{2}\;\text{dxdydz}$$

with ‖(*a*_1_, *a*_2_, *a*_3_)‖ = ‖(*b*_1_, *b*_2_, *b*_3_)‖ = 1. By introducing *c_i_* = *a_i_* − *b_i_*, Eq. 4 can be written as:

(5) $$d_{\Gamma }{\left(A,B\right)}^{2}=\begin{array}{ccc} \left(c_{0},c_{1},c_{2},c_{3}\right) & \underset{\text{const for}\;\Gamma }{\underset{︸}{\left(\begin{array}{cccc} \int 1 & \int x & \int y & \int z\\ \int x & \int x^{2} & \int xy & \int xz\\ \int y & \int xy & \int y^{2} & \int yz\\ \int z & \int xz & \int yz & \int z^{2} \end{array}\right)}} & \left(\begin{array}{c} c_{0}\\ c_{1}\\ c_{2}\\ c_{3} \end{array}\right) \end{array}$$

**Figure 1.**
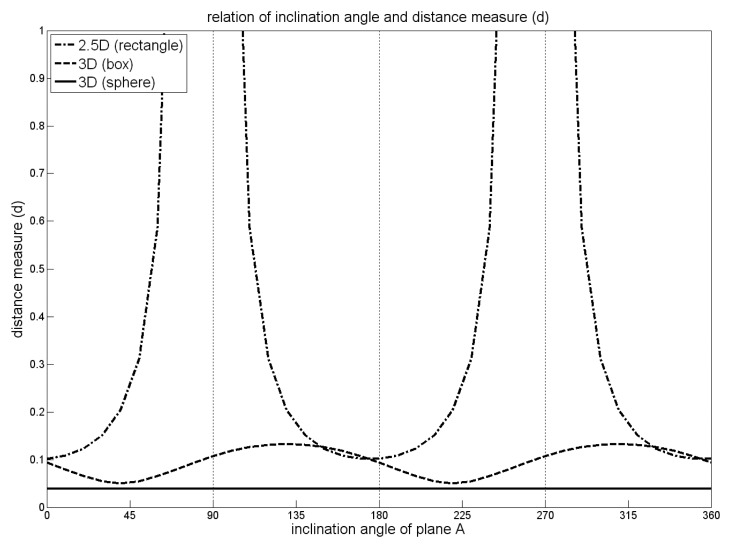
Comparison of measures d defined in 2.5D and 3D and applied to differently shaped areas of interest Γ (box and sphere). For two planes, *A* and *B*, inclined by 1^◦^) and rotated around the *y*-axis, *d* is shown with respect to the inclination angle of *A*.

If the 2.5D-distance measure *d*_Γ_(*U*, *V*) is normalized by the area of Γ, it can be interpreted as the mean distance between the two planes *U* and *V* in direction *z* within the region Γ . This measure is dependent on the coordinate system, as it increases with the angle between the normals of the considered planes and the reference direction *z* (Figure 1, dash-dotted line). [33] suggest to define different reference directions in order to fully cover the space of planes appropriately If this is done for each pair of points, the resulting distance measure computation is time consuming and it is no longer a metric. The 3D distance measure *d*_Γ_(*A*,*B*) can be interpreted geometrically, too. The integrand of Eq. 4 represents the squared difference of the orthogonal distances from a point to the two planes *A* and *B.* Thus, the integral over all squared distances within Γ can be interpreted as the mean squared distance between these planes, if it is normalized by the volume of Γ. As we consider squared differences, greater differences get a higher weight. But this does not matter, as we aim at the determination of similar planes having small differences. For a rectangular extension of Γ in the form of a bounding box in the three coordinate directions, *d*_Γ_(*A*, *B*) is not completely independent of the definition of the coordinate system. If the planes are oriented in diagonal direction with respect to the bounding faces of Γ, the measure will be greater than if the planes are parallel to a bounding face (Figure 1, dashed line). Using a spherical area of interest overcomes this, resulting in a constant measure *d*_Γ_(*A*, *B*). It is independent of the choice of the coordinate system (Figure 1, solid line), and therefore appropriate for processing 3D point clouds. The formulation as multiplication of matrices and vectors as suggested by [33] has the advantage, that the integrals are constant for a fixed Γ and the distance metric between two planes can therefore be evaluated quickly.

The segmentation itself is realized as region growing initialized by seed cluster determination in feature space. This feature space is defined by the four parameters of the local regression planes (a_0_, … a_3_) of the given points, transformed by Eq. 5. A global clustering approach in this feature space would require the evaluation of the distance measures of all pairs of given points resulting in a time complexity of O(*n*^2^), with *n* representing the number of points given. Processing millions of points in a single step would not be feasible. Therefore, the determination of the distance matrix is replaced by a sequential evaluation of the given plane parameters. This reduces the complexity of distance measure evaluation to O(*nm*) with *m* representing the number of detectable planar faces.

The seed clusters are determined in a non-parametric fashion. This can be interpreted as an analysis of the one-dimensional marginal distributions of the plane parameters making up the feature space. Initially, the parameters are discretized as histograms with one-hundred bins. The minimum and maximum values of the histograms are dependent on the distribution of the values of the given parameters. The most dominant mode of the distributions is chosen first. All points belonging to this mode are chosen and the other three marginal distributions of the chosen points are analyzed. Again, the points belonging to the most dominant mode are chosen, and the process continues, until all four plane parameter distributions are analyzed. If no dominant cluster can be determined, the number of bins is reduced by a factor of two, until at least four points are selected to define a seed cluster. If no new seed cluster can be determined, the algorithm is stopped. Algorithm 1 shows the pseudo code of this process.


**Algorithm 1**: Pseudo code of the seed cluster algorithm
**Input**: *P_xyz_* (points), *P_fsp_* (feature space parameter of points)**Output: ***PSC_xyz_* (seed cluster points)**1***nob* = initial number of bins**2****for**
*i*=*1*
**to** 4 ***do*****3** Compute histograms of *P_fsp_* with *nob* bins**4** *db* (dominant bin) = {*bin_m_* ∈ *histogram*: |*bin_m_*| = *max*(*|bin_n_|*), *n* ∈ *nob*}**5** *P_fsp_* {*P*: *P_fsp_* ∈ *db*}**6** Eliminate feature space parameter containing *db* from *P_fsp_***7****end****8****if** |*P_fsp_*| >= 5 **then****9** *PSC_xyz_* = *P_fsp_***10****else if**
*nob* < *4*
**then** return Ø**11****else**
*nob* = *ceil*(*n*/2); JUMP TO 2


**Figure 2.**
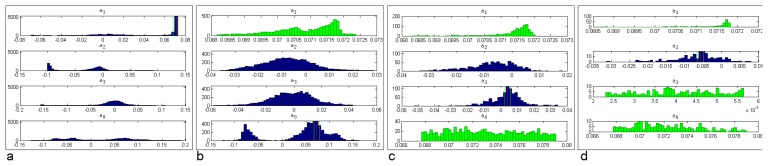
Seed cluster determination by sequential evaluation of the individual plane parameters in the feature space. (a) shows the histograms of all points (most dominant parameter: *a*_1_). The selected points (green) are analyzed further ((b) and (c)). Finally, 27 points define the seed cluster (d).

The feature space analysis for finding a seed cluster is demonstrated using a point cloud representing a set of different planes. Figure 2 shows the distributions of the regression plane parameters of the given points as histograms. The most dominant cluster is found in the marginal distribution of *a*_1_ (Figure 2a). The three other sub-figures show how the points are selected from the other plane parameters. The finally selected points define the seed cluster.

While the seed clusters are determined in feature space only, the final segments are determined considering this feature space as well as a distance threshold in object space. The points assigned to the seed cluster are used to define a seed plane, and all points in proximity to this plane are extracted. Afterwards, *d* is determined for these points with respect to the seed plane, and points with a d smaller than a threshold value are added to the segment. Finally, an adjusting plane is determined for all accepted points. This plane will differ (slightly) from the starting plane, and the process is therefore applied iteratively. If no points are added or removed, the iteration stops. The points of the segment are removed from the available points and the segmentation continues with the next seed cluster determination. Algorithm 2 shows the pseudo code of the segmentation algorithm.


**Algorithm 2:** Pseudo code of the segmentation algorithm
**Input: ***P_xyz_* (points), *P_fsp_* (feature space parameter of points), *th_os_* (distance threshold in object space)**Output**: *P_id_* (plane segment index per point), (*a*, *b*, *c*, *d*) (plane parameter)**1****repeat****2** *PSC_xyz_* =Algorithm 1(*P_xyz_*)**3** *scpl* = *nspl* = regression plane(*PSC_xyz_*)**4** **repeat****5**   
$p_{\text{newseg}}=\{PSC_{\text{xyz}}^{i}:\text{normDist}(PSC_{\text{xyz}}^{i},\text{scpl})<th_{os}\}$**6**  determine *d*(*P_newseg_*, *scpl*)∀*P_newseg_* (cf. Eq. 5)**7**   
$p_{\text{newseg}}=P_{\text{newseg}}\backslash \{P_{\text{newseg}}^{i}:d_{i}<th_{fs}\}$**8**  *nspl* (new segment plane) = regression plane(*P_newseg_*)**9**  *PSC_xyz_* = {*P*: *P_newseg_* ∈ *nspl*}**10** **until**
*scpl* = *nspl***11**  
$P_{id}^{i}=\text{new plane id}\forall P_{\text{xyz}}\in P_{\text{newseg}}$**12** *P_xyz_*=*P_xyz_* \ *P_newseg_***13****until**
*new(sc)* = ∅**14****if** ∃ *disjunct sub-segments in a segment*
**then** split segment**15****if** ∃ *similar and touching segments*
**then** merge segments


**Figure 3.**
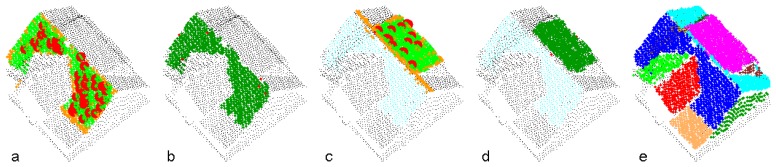
Detection of planar faces from a point cloud by segmentation. (a)-(d): Determination of the segments 1 and 2. (a) and (c): Seed cluster points (large red dots), points accepted in object space (orange), and points accepted in feature space (green). (b) and (d): Result of the robust plane fit (dark green: accepted; red: rejected). The small cyan dots in (c) and (d) represent points already assigned to segment 1. Black points have not been used so far. (e): Final segmentation including roof and wall faces.

Figure 3 demonstrates the segmentation process applied to a point cloud representing one building. The determination of the first two segments is shown in (a)-(d). In (a) and (c), the seed clusters are emphasized (large red dots), as well as the points accepted by the object space criterion (orange) and the feature space criterion (green). Points, finally assigned to the planar segments are shown in dark green in (b) and (d). The final segmentation is shown in (e).

### 3.2. Building extraction

We define a roof outline as polygonal 2D boundary of the orthogonal projection of all points representing a building onto the horizontal xy-plane. The determination of such building point clouds is realized as a combination of the previously described segmentation and a mean shift based segmentation [34]. Described shortly, the mean shift algorithm finds clusters by shifting each point to the barycenter of its neighboring points. A parameter of this algorithm is therefore the neighborhood size (spatial extent in *x*, *y*, and *z*-direction). This shift operation on all points is performed iteratively and each point moves towards a cluster center. All points assigned to a cluster center define a segment.

The building extraction is applied to point clouds representing areas containing one complete house. The estimation of such an area does not need not be very precise. Either initial points may be determined interactively, or automated seed region selection can be used (e.g. [35], [19]). Anyway, terrain, vegetation, and other objects may be part of this region, too. For each point, a local normal vector is estimated considering a fixed number of neighbors. The neighborhood size is dependent on data noise, sampling properties (e.g. sampling distance), and other data characteristics (e.g. referencing deficiencies). From this neighborhood, fifty percent plus one point are selected by a robust approach [36] to determine a local regression plane. Points with estimated normal vectors indicating steep inclination angles are excluded, because such points are interpreted as lying on (vertical) walls and not on roof faces. As we are aiming at determining connected roof faces representing individual buildings, walls are counterproductive as they are likely to connect neighboring buildings or roofs and terrain. However, for the building modeling approach, points on walls are considered.

**Figure 4.**
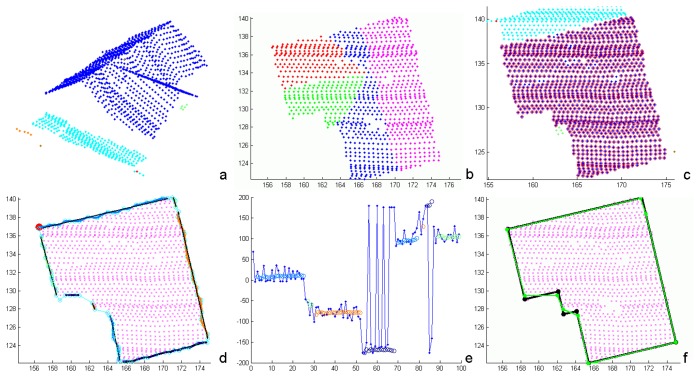
Building outline determination initiated by mean shift segmentation (a) and (c) and planar face extraction (b). The points assigned to one building are shown as red circles in (c) and as magenta crosses in (d) and (f). The generalization of the 2D *α*-shape (cyan polygon in (d)) is applied using an angular criterion (e). The generalized (green) and the regularized outline (black) are shown in (f).

The first step of the building extraction is the mean shift segmentation. Based on predefined criteria like the position of the segment within the estimated boundary or the number of points of the segment, one segment is selected as the initial roof area. The reliability of further reconstruction is increased, if roof points of exactly one house are included in and if terrain points are excluded from this initial area. The latter requirement can be supported by eliminating points near a given DTM first. After selecting the initial roof area, an iterative region growing process is started by decomposing all mean shift segments into sets of points representing planar faces. This is realized by means of the segmentation approach described in Section 3.1.. This segmentation rejects vegetation points (e.g. overlapping trees), as the local roughness of neighboring points is considered implicitly. I.e., the local normal vectors of points belonging to one planar segment are assumed to be similar with respect to the feature space distance criterion, hence having a low variance. All planar faces found by this segmentation are compared to those of the initial segment and if two faces are similar, the respective mean shift segment and the initial roof area are merged. Subsequently, the planes of the remaining segments are compared to those of the merged segment. This process is repeated, until no more segments can be merged. Finally, the roof outline is generated using all points assigned to one building. As a byproduct, these points are assigned to planar faces as well. These planar faces represent individual roof faces and are used for the modeling process. Figure 4 shows the mean shift segmentation of a point cloud (a), the planar faces segmentation (b), and the points assigned to one building (red circles in (c), superimposed to the mean shift result).

### 3.3. Roof outline generation and regularization

The roof outline generation is initialized by the computation of a 2D *α*-shape [37] of all building points. A 2D *α*-shape is a polygonal boundary of a point set. Its shape depends on the *α* value which has to be chosen carefully or estimated properly from the data, as for too large values, the *α*-shape result is the convex hull of the point set. This would prevent a proper representation of concave outlines, as occurring, e.g., in the case of L-shaped buildings. Values in the order of two times the mean linear point distance produce reliable building outline polygons, also representing small details. However, these polygons consist of rather short and irregular line segments (cyan polygon in Figure 4d). To determine more regular building outlines, a post processing of the *α*-shape polygons is necessary.

We apply a generalization based on the detection of connected, linear components within the *α*-shape. For this, we analyze the angular direction of subsequent line segments of the *α*-shape. Starting from the initial point of the *α*-shape polygon (red dot in Figure 4d), the angular direction of the first polygon segment is determined. The subsequent segment is assigned to the same linear component, if the angular deviation between the two components is smaller than a threshold. Afterwards, the mean angular direction is determined and the angular deviation to the subsequent polygon segment is analyzed. This process is stopped, if the deviation is larger than the threshold. The angular deviations of subsequent segments are shown in Figure 4e (blue dots). The mean directions are superimposed as circles, using the same color coding as in (d). The final parameters of the linear components are then determined by 2D orthogonal distance regression lines for the respective *α*-shape vertices.

These linear components are subject to a regularization, enforcing orthogonality or parallelism between them. The adequate condition is estimated from the data. The parameters of the boundary lines are determined by means of a simultaneous adjustment with individual distances of the lines to the origin, but only one orientation parameter. Hence, the adjusted lines are either parallel or orthogonal to this one orientation. The initial orientation is taken from the longest boundary line segment. A regularized boundary line is accepted, if the angular deviation before and after regularization is below a threshold value. Otherwise, the original boundary line is maintained. If subsequent components are forced to be parallel, they are either merged or an intermediate, orthogonally oriented segment is inserted. The final building outline is obtained by the intersection of the line segments. Figure 4f shows the generalized *α*-shape polygon (green) and the final, regularized building outline (black).

### 3.4. Model generation

We define a building model as a composition of a set of planar faces, which shall be the best approximation of the given point cloud. Generally, these faces can be categorized as terrain intersection faces, wall faces, or roof faces. According to the definition of *CityGML* [38], the Terrain Intersection Curve (TIC) is the “*exact position where the terrain touches the 3D object*”. Assuming a building model with vertical walls intersecting the roof model at its eaves and thus not modeling roof overhangs, the building outline, projected onto the DTM, represents the TIC. Otherwise, if roof overhangs are modeled properly, the building outline as defined in Section 3.2. represents the projection of the eaves of the roof onto the DTM and therefore differs from the TIC. The terrain intersection faces are determined by triangulating the points of the TIC while considering the edges of the TIC as forced edges. Wall faces may either be determined by means of points (if enough points are available) or according to certain assumptions. In general, we define walls as vertical faces above the edges of the building outline and intersecting the roof segments situated above those edges along the eaves.

The most challenging process is the modeling of the individual roof faces representing the roof. We define a roof face as the closed, polygonal boundary of a roof segment. Such a segment is determined by the point cloud segmentation (cf. Section 3.1.). It represents, per definition, one planar face of the respective roof. An edge of such a polygonal segment boundary can be an intersection line of two neighboring roof segments, an intersection line of a roof and a wall segment, or a so-called “jump edge”. Typically, jump edges occur at vertical faces within the roof which are not represented properly by the point cloud.

The roof modeling process is applied sequentially to the prior determined planar roof segments. It uses a local coordinate system. This is necessary to define the alignment of faces connecting two roof segments along jump edges. A realization of such a coordinate system is the vertical projection of the points onto the horizontal *xy*-plane. The modeling process starts with the determination of segments (roof segments and wall faces) which are neighbors to the investigated segment. Afterwards, all intersection edges of the investigated segment and its neighbors are determined. The next step is the pairwise intersection of these edges. The closed polygonal boundary of the roof segment is determined, by selecting a connecting path of such intersection edges and considering two selection criteria: The position of the edges with respect to the points defining the investigated roof segment and the length of the edges. If no closed polygonal boundary can be determined by the intersection edges alone, jump edges are introduced applying the approach, described for the outline generation (cf. Section 3.3.).

## 4. Application and experiment

**Figure 5.**
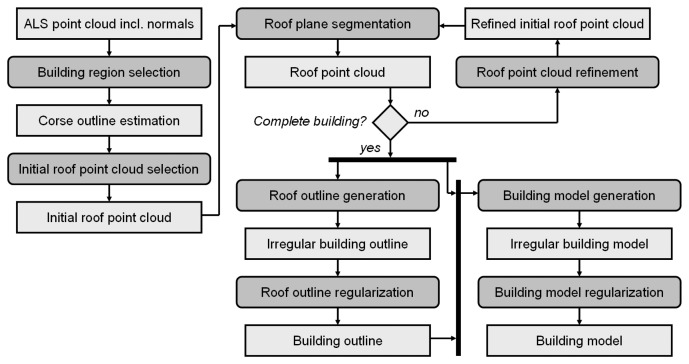
Suggested workflow from building extraction to building modeling

The proposed workflow is based on the sequential determination of individual building models from laser scanning point clouds. An activity diagram of this workflow is shown in Figure 5. Input is an unstructured point cloud. For each point, a local regression plane is estimated considering a fixed number of neighbors. Dependent on several factors (cf. Section 3.2.), neighborhood sizes between 8 and 32 points are commonly used for the robust selection of fifty percent plus one point to estimate the local regression plane. This robust approach ensures, that sharp structures (e.g. gables) are not smoothed.

In “*building region selection*” an area containing one complete building is extracted from the point cloud. As already mentioned, terrain, vegetation, and other objects like other buildings or building parts may be part of this region. However, points acquired at steep regions (e.g. inclination angle >80^◦^) are, however, excluded for the subsequent step “*roof outline generation*”. The selected point cloud is input to the “*initial roof point cloud selection*” where the mean shift segmentation (cf. Section 3.2.) is applied. The ‘initial roof point cloud’ is determined considering the positions of the mean shift segments within the estimated boundary (e.g. center, not touching the border, …) and the number of points assigned to these segments. The final ‘roof point cloud’ is determined in an iterative process. “*Roof plane segmentation*” performs a decomposition of all mean shift segments into planar faces (cf. Section 3.1.) and compares them to the ‘initial roof point cloud’. If identical planar faces are found in a mean shift segment and the initial segment, the two segments are merged. The iteration stops if no more similar planes are detected. The result of this process is a point cloud, representing one building. As byproduct of the roof plane segmentation, all points of this point cloud are assigned to planar faces, representing individual roof or wall faces.

This segmentation is used as input to the modeling approach, and all points assigned to planar regions are used to generate the outline of the building (“*roof outline generation*”). In contrast to the unique outline that can be determined from raster data, numerous polygonal outlines of an irregular point cloud can be determined. Our outline generation is initialized by the determination of a 2D *α*-shape which is subsequently generalized and regularized (cf. Section 3.3.).

**Figure 6.**
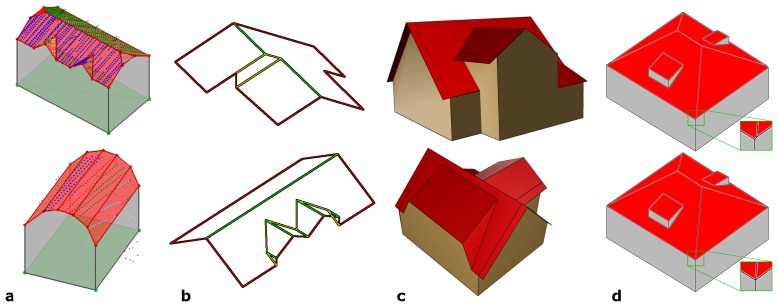
Different representations of 3D models of buildings with complex roof structures. (a): Waterproof model and color coded segmentation result; (b): Edge-model (red: eaves, green: gables, yellow: other edges); (c): 3D model with roof overhangs; (d): Roof model regularization.

The regularized building outline and the segmentation result of the ‘roof point cloud’ are input to the “*building model generation*”. This approach is based on the determination of polygonal boundaries of each planar face. Faces at so called ‘jump edges’, connecting the building boundary and the eaves, but occurring in several cases within the roof of a building as well, are – by definition – vertical. Ideally, a closed roof face boundary can be composed of eave edges and intersection edges of neighboring faces. In this case, it is sufficient to determine the neighborhood topology of the current roof face considering its neighboring faces and its neighboring outline edges, only. If this is not the case, e.g. if jump edges do occur, the *α*-shape based approach, already described for the outline detection, is applied to determine the missing edge segments. Figure 6 shows automatically determined building models. In (a), models of a building with small details (upper) and of a building with a cylindrical roof (lower) are shown in transparent rendering. The segmentation of the original point clouds are superimposed color coded. (b) shows highly detailed roofs determined from high density ALS point clouds. The edges are classified in eaves (red), gables (green), and other edges (yellow). (c) shows a building model with a set of properly modeled walls and roof overhangs, as, for some vertical faces, enough points were available (cf. Figure 3).

The final “*building model regularization*” investigates corners of the building model. Unambiguous corner points can be identified, if three intersecting planes are involved. In many cases, however, building corners along the eaves are intersections of more than three planes (e.g. two walls and two roof faces) resulting – in general – in several intersection points of sets of three faces, situated close to each other. To cope with this problem, we analyze the distances between all intersection points and merge them if the distance is smaller than a certain threshold. Figure 6d shows the original model (upper) and the regularized one (lower row). It has to be noted, that this operation overcomes the assumption for planar roof faces, as the regularized roof polygons no longer fulfill the planarity criterion.

## 5. Result and discussion

So far, we demonstrated the behavior of the individual processing steps of our approach. To demonstrate the applicability of the proposed workflow for the automated processing of huge areas – as required for the generation of 3D city models – we show results of several projects and compare them, if possible, to independently determined reference data. Additionally, we discuss advantages of this approach, compared to results of a model driven one. It has to be noted, that there is only little literature on the question of how to determine the quality of a city model. For example, in [39], points acquired by a tacheometer were used as reference to compare the accuracy of city models, and in [35], the validation of building outlines is discussed. In terms of a quality assessment, these approaches are regarded as partial, because primarily deviations are described by statistical means whereas completeness or level of detail remain unconsidered. We assumed that our modeling approach results in a composition of planar faces, aiming at approximating the building point cloud best (cf. Section 3.4.). Therefore, we suggest to validate building models by comparing them to the given point clouds.

**Figure 7.**
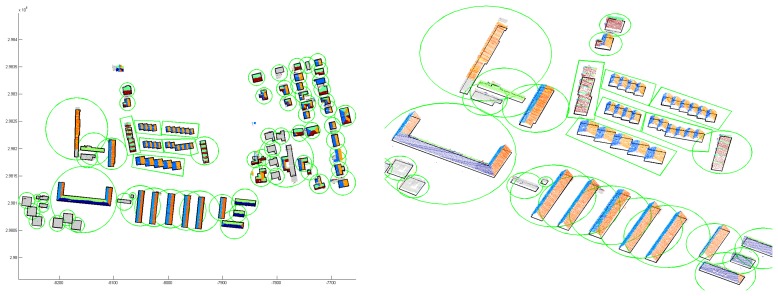
Result of building outline extraction. Building region estimation (green), extracted building outline (black), and original points (color: local aspect angle, gray: horizontal).

The first example (Figure 7) shows the result of an outline extraction applied to a dataset acquired at a suburban area by a Riegl LMS Q560. It contains several huge apartment buildings (up to 60 m length), a set of row houses, and several small buildings. The mean point density is approximately 6 points per m^2^ and points close to the DTM were eliminated prior to the outline detection. The building regions were estimated by building center points and radii of circles containing complete buildings. Although, these estimates are very coarse and even overlapping, the automated outline extraction worked properly.

The second dataset was acquired at a rural settlement with individual buildings. Again, a Riegl LMS Q560 was used for data acquisition. The mean point density is similar to the first example, and points close to the DTM were eliminated as well. The coarse building outlines were digitized interactively.

**Figure 8.**
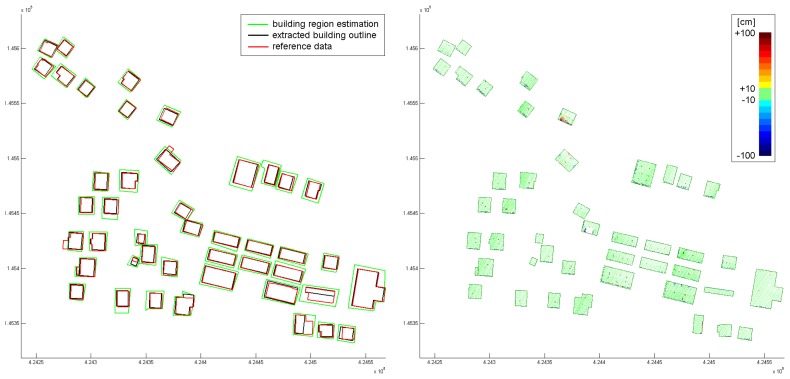
*Left*: Analysis of building outline extraction (green: coarse estimation of the building region, black: extracted building outline, red: reference data). *Right:* Distances of original points with respect to the regression planes representing the roof segments.

Figure 8 (left) shows the estimated outlines (green), and the finally extracted and regularized outlines (black). Additionally, results from an independent stereo-analysis of areal images were available (red). Figure 8 (right) shows color coded orthogonal distances of the points used for the building modeling with respect to the determined roof faces. It can be seen clearly, that in most areas, the point cloud is well approximated by the building models. Only very small structures (e.g. chimneys) are not modeled properly. Two buildings in the center area show severe differences.

**Figure 9.**
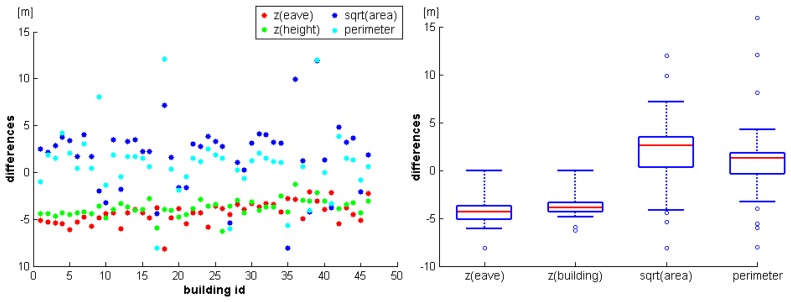
Comparison of parameters determined for individual buildings from the ALS point cloud and from independent reference data. *Left:* Differences of eave height, building height, building area (square root), and building perimeter are shown for each building (Difference: Reference minus ALS). *Right:* Box plots representing the same values.

Additionally, reference values of the heights of the eaves and the gables, and the areas and perimeters of the outlines were available for the buildings. Figure 9 (left) shows the differences of the reference values minus the values determined from the ALS data. For scaling reasons, the square root was computed for the building area differences while the other values are already given in meters. The differences determined for the two height attributes (heights of eaves and buildings) are very similar for different houses. However, there is a detectable trend of approximately minus five meters, indicating a possibly wrong referencing of one of the two datasets. Additionally, this trend seems to decrease from building 1 to building 46. As the buildings were processed from East to West, this might indicate a non constant offset between the two datasets. Except for some outliers, the differences of the perimeter and the area attributes are small. However, again there seems to be a small, systematic difference, as the ALS buildings are slightly smaller. Figure 9 (right) shows the same results as boxplots (red lines: medians, boxes: 25 to 75% quantils, minimum and maximum whiskers: 95% confidence intervals, circles: outliers).

**Figure 10.**
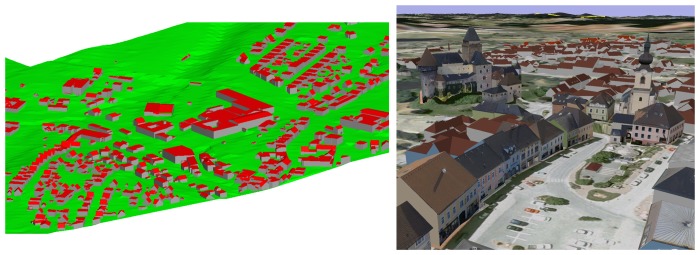
*Left:* Rendered city model correctly intersected with a DTM triangulation. *Right:* 3D city model with manually applied textures from terrestrial and airborne images.

The third example (Figure 10) shows a model of a small city consisting of approximately 2000 buildings. The data comprises several flight strips and was acquired by a Riegl LMS Q560. The mean point density of approximately 20 points per m^2^ is, compared to the other two examples, much higher. In this example, the exact position of the outlines was determined interactively from the ALS data by digitizing. After the automated data processing, possibly erroneous buildings were indicated for further, interactive post processing by means of a statistical analysis of the resulting building models. In this post processing step, an interactive module was used to validate and, if necessary, edit the segmentation result. If the resulting model was still incorrect, it was finalized using a commercial CAD software. Figure 10 (left) shows a part of the final city model. The roofscape is shown in red and the vertical walls in gray. The triangulated terrain model, determined from the same ALS point cloud and correctly intersected with the walls at the terrain intersection curve (cf. Section 3.4.) is shown in green. Figure 10 (right) shows the final model as a rendered 3D city model. The texture maps have been acquired by airborne photogram-metry (roofs) and from terrestrial view points (facades). They were mapped onto a 3D triangulation of the city model.

**Figure 11.**
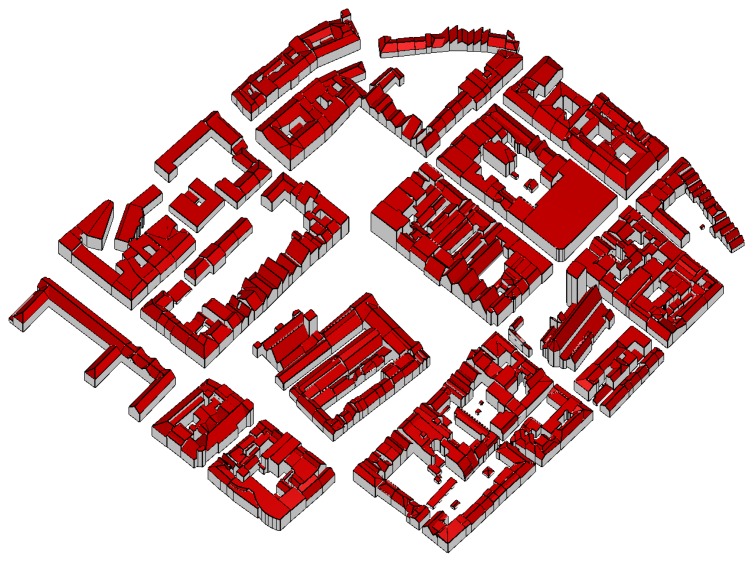
Model of a historical city determined by the proposed, data driven approach. The roofscape is shown in red and vertical walls in gray.

**Figure 12.**
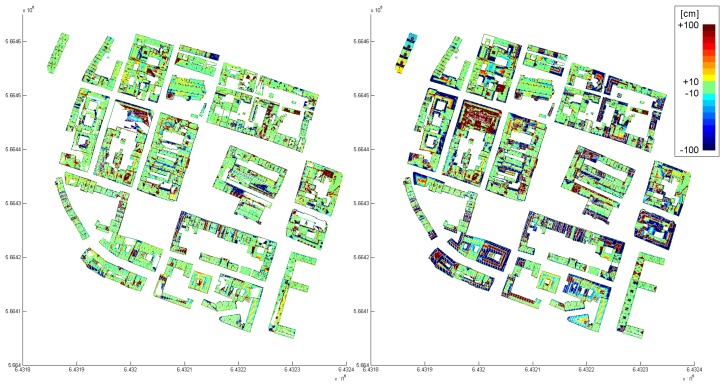
Visual comparison of city models which were automatically determined from the same data set (cf. Figure 11), but by applying two different approaches (*Left*: proposed data driven approach; *Right*: model driven approach). Distances of the original points with respect to the planes defining the roof segments are shown color coded.

By means of the final example, we compare – visually – a model, determined by the proposed data driven approach, to a model, determined by a model driven approach. This model driven approach uses a set of predefined building outlines (rectangular, L-, T-, or U-shaped) and a set of roof models (e.g. saddle roof, hip roof, …) to reconstruct the individual buildings. If the model catalog does not fit to the point cloud, a block model is generated. Figure 11 shows the city model, determined by the proposed, data driven approach and finalized interactively. The data was acquired by an Optech ALTM 2050 and the mean point density is approximately 4 points per m^2^. The differences of the automatically determined model with respect to the original point cloud are shown color coded in Figure 12 (left). The color coding is equal to that, used in Figure 8 (right). The model, determined by the model driven approach was analyzed, using the same difference computation. The color coded differences are shown in Figure 12 (right). Comparing these two difference visualizations, the advantages and disadvantages of the two approaches become visible. While the data driven approach obviously approximates the original point cloud better (much more green regions), the model driven approach has a higher completeness (no white areas within the buildings). However, in many cases, significant structures of the roofs are neglected by the model driven approach.

To ensure a high degree of automatism, as few parameter as possible are to be determined interactively. As described in Section 3., two thresholds are necessary for the proposed approach: A distance criterion in feature space and a distance criterion in object space. However, as the feature space distance criterion is applied to a normalized region of interest (i.e. the unit sphere), it is not dependent on the extension of the object to be investigated. The object space criterion is – in general – correlated to the error budget of the data. In principle, this threshold has to be chosen as small as possible. However, as the point clouds in general comprise certain random and systematic errors, this threshold has to cope with such deficiencies. But, it can be estimated from the data, by estimating the variance of the data points with respect to planar faces. As mentioned in Section 3.2., another parameter to be set is the neighborhood size for the point wise normal vector estimation. However, this parameter is correlated to the error budget of the data as well, and can, therefore, be estimated from the data in the same way.

So far, we suggested concepts to evaluate the quality of huge models. However, for the automated evaluation of the quality of individual buildings or building parts, more detailed criteria are required. We apply the following proofs to decide if a building is modeled properly or not. First, a check is performed if all planar segments found by the segmentation are represented by closed, polygonal boundaries. Second, for each roof plane, the area of the point segment represented by its α-shape boundary is compared to the area of the polygonal model boundary. And finally, the height values of the points defining a segment and of the polygonal model boundary are compared. If all these criteria are plausible for all faces representing a building, the building is indicated to be properly and completely modeled. Otherwise, the building is indicated for further, interactive post processing. But, in general at least some roof faces are modeled properly for such buildings, hence, supporting the interactive finalization. According to these criteria, for typical projects like the presented, the suggested algorithm allows modeling about 75% of the buildings completely. For another 15% of the buildings, more than 50% of the roof faces are modeled properly.

## 6. Conclusions

We presented a comprehensive approach for the determination of 3D building models from ALS (resp. LiDAR) point clouds. To ensure completeness, it is advisable to initialize the very first step, namely the coarse selection of building regions, interactively. All subsequent steps (i.e. outline extraction and regularization, planar faces detection, building model generation, and finally intersection with a DTM) are applied automatically. By means of a statistical analysis of the resulting building models, possibly erroneous buildings are indicated and can be improved by interactive post processing.

Our approach is data driven. Hence, the resulting models aim at approximating the given point clouds best. Compared to other, so called model driven approaches, a data driven approach is more flexible. While for model driven approaches, the possible variety of the resulting models is restricted by a predefined catalog of buildings or building parts, a data driven approach requires certain, in general more flexible assumptions, only. A typical assumption is – as in our case – that buildings can be modeled by a composition of planar faces. This might be a restriction, considering curved roof structures. But, as demonstrated, even a cylinder can be approximated properly by a set of planar faces.

The portion of completely properly modeled buildings is about 75%. However, from the remaining buildings many roof faces are modeled properly. Therefore, an interactive finalization of the city model can be done efficiently by means of commercial CAD software. The comparison of the models to independently determined building outlines and other attributes (e.g. height) has shown, that a high correlation between the reference data and the automatically determined models does exist. The comparison of the models and the original point clouds also showed reliable results.

The proposed approach has been applied to a set of actual project data from different areas and representing different types of settlements. Furthermore, the data sampling rates of the available data sets and their quality with respect to to their error budged differed. Nevertheless, the results met the requirement of being accurate and reliable. Subsequent processing like texture mapping was possible. The resulting models are well structured and topologically correct and are, therefore, directly applicable for city management processes.
